# Supplementary material for: Ubiquitin and SUMO conjugation as biomarkers of acute myeloid leukemias response to chemotherapies
Source: Life Sci Alliance. 2020 Apr 17;3(6):e201900577. doi: 10.26508/lsa.201900577 (PMC7167290; doi:10.26508/lsa.201900577)
Supplement: Supplementary file 6 [file LSA-2019-00577_Supplemental_Data_1.zip › supplementary methods - R code/Genetic Algorithm.docx]

####################################################################

## Script to select best variables for a classification mode using genetic algorithms.

## Based on `GA` library with custom fitness function.

## This script is explained in the post:

## https://blog.datascienceheroes.com/feature-selection-using-genetic-algorithms-in-r/

####################################################################

# Install packages if missing

list.of.packages <- c("parallel", "doParallel", "caret", "randomForest", "funModeling", "tidyverse", "GA")

new.packages <- list.of.packages[!(list.of.packages %in% installed.packages()[,"Package"])]

if(length(new.packages)) install.packages(new.packages)

# Load libraries

library(caret)

library(randomForest)

library(funModeling)

library(tidyverse)

library(GA)

source("lib_ga.R")

data=read_delim("data_breast_cancer2.csv", delim = ",")

# Data preparation

data2=na.omit(data) # <- use with care...

data_y=as.factor(data2$diagnosis)

data_x=select(data2, -diagnosis, -id)

# GA parameters

param_nBits=ncol(data_x)

col_names=colnames(data_x)

# Executing the GA

# Executing the GA

ga_GA_1 = ga(fitness = function(vars) custom_fitness(vars = vars,

data_x = data_x,

data_y = data_y,

p_sampling = 0.7), # custom fitness function

type = "binary", # optimization data type

crossover=gabin_uCrossover, # cross-over method

elitism = 3, # number of best ind. to pass to next iteration

pmutation = 0.03, # mutation rate prob

popSize = 50, # the number of indivduals/solutions

nBits = param_nBits, # total number of variables

names=col_names, # variable name

run=5, # max iter without improvement (stopping criteria)

maxiter = 50, # total runs or generations

monitor=plot, # plot the result at each iteration

keepBest = TRUE, # keep the best solution at the end

parallel = T, # allow parallel procesing

seed=84211 # for reproducibility purposes

)

# Checking the results

summary(ga_GA_1)

# Following line will return the variable names of the final and best solution

best_vars_ga=col_names[ga_GA_1@solution[1,]==1]

# Checking the variables of the best solution...

best_vars_ga

# Checking the accuracy

get_accuracy_metric(data_tr_sample = data_x, target = data_y, best_vars_ga)

custom_fitness <- function(vars, data_x, data_y, p_sampling)

{

# speeding up things with sampling

ix=get_sample(data_x, percentage_tr_rows = p_sampling)

data_2=data_x[ix,]

data_y_smp=data_y[ix]

# keep only vars from current solution

names=colnames(data_2)

names_2=names[vars==1]

# get the columns of the current solution

data_sol=data_2[, names_2]

# get the roc value from the created model

roc_value=get_roc_metric(data_sol, data_y_smp, names_2)

# get the total number of vars for the current selection

q_vars=sum(vars)

# time for your magic

fitness_value=roc_value/q_vars

return(fitness_value)

}

get_roc_metric <- function(data_tr_sample, target, best_vars)

{

# data_tr_sample=data_sol

# target = target_var_s

# best_vars=names_2

fitControl <- trainControl(method = "cv",

number = 3,

summaryFunction = twoClassSummary,

classProbs = TRUE)

data_model=select(data_tr_sample, one_of(best_vars))

mtry = sqrt(ncol(data_model))

tunegrid = expand.grid(.mtry=round(mtry))

fit_model_1 = train(x=data_model,

y= target,

method = "rf",

trControl = fitControl,

metric = "ROC",

tuneGrid=tunegrid

)

metric=fit_model_1$results["ROC"][1,1]

return(metric)

}

get_accuracy_metric <- function(data_tr_sample, target, best_vars)

{

data_model=select(data_tr_sample, one_of(best_vars))

fitControl <- trainControl(method = "cv",

number = 3,

summaryFunction = twoClassSummary)

data_model=select(data_tr_sample, one_of(best_vars))

mtry = sqrt(ncol(data_model))

tunegrid = expand.grid(mtry=round(mtry))

fit_model_1 = train(x=data_model,

y= target,

method = "rf",

tuneGrid = tunegrid)

metric=fit_model_1$results["Accuracy"][1,1]

return(metric)

}
